# Supplementary material for: Are carbon emissions trading and green financial instruments synergistic? -Comprehensive quantitative research based on content analysis
Source: PLoS One. 2024 Mar 7;19(3):e0298601. doi: 10.1371/journal.pone.0298601 (PMC10919601; doi:10.1371/journal.pone.0298601)
Supplement: S1 Data — (ZIP) [file pone.0298601.s001.zip › Data/地级市Z_F工作报告.zip!/║╙─╧/─╧╤⌠╩╨/└┤╫╘╣1⁄2╓┌║┼í╛┬φ┐╦╩2╛▌═°í┐_2010.docx]

各位代表:

现在，我代表市人民政府，向大会作工作报告，请予审议，并请各位政协委员和其他列席人员提出意见。

折叠编辑本段工作回顾

一、去年工作回顾

2009年是近年来我市经济社会发展最为困难的一年。面对严峻的经济形势和繁重的发展任务，全市上下深入贯彻科学发展观，立足于保增长、保民生、保稳定，坚定信心，抢抓机遇，锐意进取，扎实工作，较好地完成了市四届人大一次会议确定的各项目标任务。

(一)全力战危机保增长，经济实现平稳较快发展。全面落实中央、省应对国际金融危机的一系列决策部署，抓住经济运行的关键环节，决战二季度、大干三季度、决胜四季度，保持了经济平稳较快发展的良好势头。预计全市生产总值完成1780亿元、同比增长11%，地方财政一般预算收入56.2亿元、增长9.5%。深入开展"企业服务年"活动，规模以上工业增加值逐月回升，全年完成486.4亿元、增长14%，实现利润96.2亿元、增长9.6%。深入开展"项目推进年"活动，城镇固定资产投资完成929.5亿元、增长31.2%，其中工业投资603亿元、增长36.4%。全年投资3000万元以上新开工项目达1132个，是上年的4.3倍。市级重点项目完成投资230亿元，42个项目建成投产，75个项目开工建设。积极发展第三产业，认真落实国家鼓励扩大消费的政策，全社会消费品零售总额完成676.7亿元、增长19%。

(二)重视加强"三农"工作，农业和农村经济稳步发展。全面落实各项支农惠农政策，全市发放各项涉农补贴资金25.5亿元。粮食生产在大灾之年再获较好收成，总产达115.9亿斤。油料总产111万吨，增长9.2%;烟叶产量5.9万吨，增长8%;肉蛋奶总量121.3万吨，增长6.2%。完成工程造林130万亩，是历年来生态造林最多的一年。龙大牧原年屠宰加工100万头生猪、科尔沁牛业年屠宰加工10万头肉牛、三色鸽豆业等农业产业化项目建成投产，新发展农民专业合作社654家。农村基础设施建设扎实推进，新建改建农村公路2361公里、改造危桥1万余延米，新增农村沼气用户10.2万户，解决47.3万农村居民安全饮水问题，巩固和解决贫困人口12.3万人。完成200个试点村、54个示范村、1782个村容村貌整治村建设任务。

(三)加大城镇建设管理力度，城镇面貌明显改观。中心城市基础设施建设步伐加快，完成了3条道路大修改造和54条背街小巷改造任务，独山大道南延、仲景中路快车道、机场新航站楼建成投运，环城高速全线贯通，2条道路改扩建、4条新建道路、仲景大桥、污水处理厂二期、梅溪河综合整治示范段、滨河路污水管网建设、南阳机场二期改扩建等工程进展顺利，城中村、旧城改造稳步推进。新增城市绿化面积21万平方米，新投放出租车近300辆、公交车100辆。城市建设全年新开工项目总投资突破20亿元。城市管理体制进一步理顺，39项城市规划建设管理权限下放或委托给三区。"六创一迎"活动成效显著，城区交通秩序有所改进，环境卫生、市场秩序、市容市貌明显好转，违法建设和工程建设领域突出问题得到清理整治。县城和小城镇竞相发展，亮点纷呈。全市城镇化率达到36.6%，提高1.7个百分点。

(四)着眼长远打基础，发展后劲进一步增强。扎实推进"一个载体、三个体系"建设，着力培育竞争新优势。全市13个省定产业集聚区规划通过省专家组评审，基础设施建设不断加强，入驻一大批项目。完成第二次全国土地调查和第二轮土地利用总体规划修编。围绕构建现代产业体系，委托国内知名院所分别编制我市现代产业、现代服务业、现代农业发展规划及7个重点产业、13家重点企业发展规划和农产品加工等专项规划;组织实施105个重大工业结构调整项目和15个转型升级项目，13家企业和15个重点项目进入省"双百"计划。围绕构建现代城镇体系，修订完善中心城市总体规划，面向国际征集白河两岸区域城市设计，完成一批专业规划、综合整治规划和设计方案;重视加快中心城区经济发展，启动鸭河、官庄工区筹备前期工作;完成一批县城总体规划修编和村镇体系规划。加快构建自主创新体系，全降解塑料、纤维乙醇、多晶硅、光学引擎等高新技术产品研发和产业化生产进展顺利，南阳光电高新技术产业化基地通过国家认定，4个项目列入国家"863"计划，新增国家高新技术企业5家、国家创新型试点企业2家，蝉联全国科技进步先进市。节能减排超额完成省定目标。

(五)深入推进改革开放，发展活力不断增强。市属国有工业企业改革继续深化。商贸流通企业改革稳步推进。投融资体制改革力度加大，整合组建了市投资集团、金地投资开发公司等投资公司。水利工程管理单位体制改革顺利完成。农村信用社改革迈出新步伐。集体林权制度改革大头落地。政府还贷二级公路收费站撤站工作如期完成。市县两级政府机构改革稳妥推进。镇平玉文化改革发展试验区规划建设走在全省前列。医药卫生体制改革启动实施。第二次全国经济普查圆满完成。中光学、金光数显、天冠、英宝电子、南防等企业分别与长虹、首钢控股、国开投、创维、平安集团进行合作。市政府分别与省建行、省中行、省工行、平安集团签署了战略合作协议。年末全市金融机构各项贷款余额699亿元，增长26.9%。实际利用外资1.33亿美元，增长12.3%;引进省外资金135.3亿元，增长22.4%。

(六)着力抓好"四大工程"，带动效应初步显现。南水北调中线工程建设进展顺利，渠首枢纽工程开工建设，水库水质稳定在Ⅱ类标准;把移民工作作为一项事关全局的政治任务来抓，库区1.08万试点移民实现了安全和谐搬迁，第一批6.5万移民安置工作正按计划进行，第二批8.6万移民安置工作顺利启动。第七届全国农运会筹备工作扎实推进，场馆区域拆迁按时完成，游泳馆、综合训练馆、主体育场开工建设，新闻中心、拆迁安置房奠基。南阳粮食主产区规划建设开始启动，水利设施建设和中低产田改造步伐加快。南阳核电项目前期进展顺利，各项可研专题已基本完成，中核河南核电有限公司在南阳注册筹建。

(七)致力改善民生，和谐社会建设稳步推进。省、市"十大实事"全面完成。城镇居民人均可支配收入实际增长10.2%，农民人均纯收入实际增长7.7%。新增城镇就业10.5万人，"零就业家庭"动态归零，城镇登记失业率控制在3.8%以内;新增农村劳动力转移就业13.5万人，劳务输出总量达235万人。企业养老、城镇职工及居民基本医疗保险覆盖面扩大;新农合参合率96%，在全省率先建立了农民就诊"一卡通"和定点医院直补制度。城乡低保、农村五保人员救助水平进一步提高，累计发放保障资金6.9亿元。经济适用住房竣工20.2万平方米，廉租住房保障1.5万余户。教育惠民政策全面落实，中小学校舍安全工程、中心城区中小学校改扩建工程和职教攻坚计划顺利推进。年度文化惠民工程圆满完成。城乡医疗卫生条件进一步改善。甲流感、手足口病等传染病防控成效显著。全民健身活动蓬勃开展，成功举办了市三运会暨首届农民运动会。人口自然增长率控制在5.07‰以内，荣获全国人口和计划生育综合改革示范市。应急建设和政府信息公开工作得到加强。安全生产形势持续好转，事故起数和死亡人数明显下降。认真做好信访工作，切实解决群众合理诉求;推进平安南阳建设，社会大局保持和谐稳定。切实加强精神文明建设，公众道德水平和城乡文明程度得到提升。

人事、司法、工商、税务、质监、海关、检验检疫、气象、地震、住房公积金管理、外事、侨务、对台、民族宗教、残疾人以及国家安全、国防教育、民兵预备役、"双拥"等各项工作都取得了新的成绩。驻宛解放军和武警部队在支持地方建设、维护社会稳定等方面作出了重要贡献。坚持依法行政，自觉接受人大及其常委会监督，支持政协参政议政，办理人大代表建议和政协委员提案855件，办结率100%，满意率96.5%。持续推进政府机关"两转两提"和廉政建设，强化行政监察和审计监督，政风行风进一步好转。

总结过去一年的工作，我们遇到的困难比预想的要多，取得的成绩比预想的要好。体会比较深的有以下几点:一是坚持把科学发展观贯穿于政府工作的各个领域、各个环节，扎实做好打基础、增后劲、管长远的工作，注重规划引领，强化项目支撑，大力推动结构调整和发展方式转变，努力实现全面协调可持续发展。二是坚定不移地贯彻落实中央、省各项政策，结合南阳实际，根据形势变化，及时调整经济运行协调服务的方向和重点，环环相扣，锲而不舍，强化措施，促使经济运行逐月回升，实现平稳较快增长。三是始终把保障和改善民生放在突出位置，集中力量，切实解决就业、就学、就医、吃水、住房、出行和社会保障等关系群众切身利益的问题，让改革发展成果更多地惠及于民。四是积极推进改革开放和体制机制创新，致力破解发展难题，经济社会发展动力和活力进一步增强。五是深入开展"两转两提"，健全机制，落实责任，高标准要求，快节奏推进，重在运作，求实求效，不断提高政府执行力。六是坚持以大局统一思想，以事业凝聚人心，变压力为动力，化挑战为机遇，迎难而上，团结奋斗，成功完成了南阳历史上规模最大、时间最紧、任务最重、政治性最强的移民搬迁试点和农运会场馆用地拆迁两大艰巨任务，各级各部门大局意识、责任意识和协作意识明显增强，广大涉迁群众"顾全大局、服从大局"和"舍小家、顾大家"的精神可歌可泣。

各位代表，成绩来之不易，经验弥足珍贵。这得益于省委、省政府和市委的坚强领导，得益于市人大、政协的监督支持，凝聚着全市人民及社会各界的智慧、心血和汗水。在此，我代表市人民政府，向全市广大干部群众，向驻宛单位、驻宛部队、武警官兵，向所有关心支持南阳发展的各界人士，表示崇高的敬意和衷心的感谢!

在总结成绩的同时，我们也清醒地看到当前经济社会发展面临的困难和问题:经济回升的基础尚不牢固，还存在许多不确定因素，保持经济平稳较快增长的任务仍很艰巨;工业不强、三产滞后、现代农业发展缓慢，中心城市辐射带动能力弱，经济结构不优，发展方式粗放，资源环境约束趋紧，面临加快发展速度与转变发展方式的双重压力;经济实力大而不强，人均财政收入低于全省平均水平，社会事业历史欠帐较多，公共服务、民生改善与广大群众期盼仍有较大差距;政府自身建设仍存在一些问题，部分领导干部和工作人员思想观念陈旧、干事创业意识不强、工作效率不高，有的甚至存在奢侈浪费、以权谋私、腐化堕落等现象。对此，我们一定高度重视，认真加以解决。

折叠编辑本段总体要求和任务

二、今年政府工作总体要求和目标任务

2010年是确保圆满完成"十一五"规划的最后一年，做好今年经济社会发展工作，对于夺取应对危机全面胜利、为"十二五"规划启动实施奠定良好基础至关重要。总体上看，今年宏观发展环境将好于上年，我市经济呈现较好的发展趋势、态势和气势，南阳独特的区位、市场、资源、环境等优势不断强化，南水北调中线工程建设、第七届全国农运会筹办、宁西铁路复线建设等重大工程的巨大带动作用日益显现，加快发展的条件更加充分。尽管我们面临宏观环境复杂多变、自身矛盾困难重重、区域竞争更为激烈等严峻挑战，只要我们时刻保持冷静头脑，增强机遇意识、忧患意识和责任意识，坚定信心，奋力拼搏，就一定能把各项事业推向前进，不断开创南阳经济社会发展新局面。

今年政府工作的总体要求:认真落实中央、省委、市委经济工作会议精神，深入学习实践科学发展观，坚持重在持续、重在提升、重在统筹、重在为民，积极实施项目带动、品牌带动、创新带动、服务带动和七项"行动计划"，着力推动经济发展方式转变和经济结构调整，着力加快工业化、城镇化、农业现代化进程，着力推进改革开放，着力改善民生和促进社会和谐稳定，努力保持跨越发展的基本态势，确保全面完成"十一五"规划。

今年经济社会发展的主要目标:生产总值增长11%;地方财政一般预算收入增长10%;全社会固定资产投资增长25%，城镇以上固定资产投资增长30%;社会消费品零售总额增长15%;外贸出口增长11%;实际利用境外资金增长15%;居民消费价格涨幅控制在3%左右;城镇居民人均可支配收入增长7%，农民人均纯收入增长6%;人口自然增长率控制在6.5‰以内;城镇登记失业率控制在4.5%以内;单位生产总值能耗降低6.8%，化学需氧量、二氧化硫排放量分别控制在6.51万吨和6.5万吨以内。

围绕上述总体要求和目标任务，重点做好以下五个方面的工作:

(一)加快新型工业化进程，努力转变经济发展方式

实施现代产业发展规划，大力推进产业结构战略性调整和二三产业协调互动发展，加快新型工业化进程和发展方式转变。

推进工业结构优化升级。坚持工业强市战略，加快实施各项产业发展规划。壮大战略支撑产业。以装备制造、纺织、油碱化工、冶金建材、食品为重点，优化资源配置，推进集聚发展，加快技术改造，发展高端优势产业群，实现产业和产品结构升级。培育战略性新兴产业。以新能源、生物、医药、光电、新材料为重点，加大产业链前端产品研发和后端推广应用支持力度，加快国家新能源高技术产业基地、国家光电高新技术产业基地、省生物产业高技术基地建设。依托天冠、中源化工、迅天宇、中光学、二胶、中南金刚石、宛西制药、福森药业、首控光电等重点企业，大力发展高技术产业和生态环保型企业，争取在全降解塑料、纤维乙醇、太阳能级多晶硅、数字投影系统、光电设备信息材料等产业化、规模化生产上实现突破。实施工业"双百工程"。把大型企业集团化作为产业结构调整的重中之重来抓，筛选确定100家重点企业实行动态管理，通过技术创新、战略重组、上市融资、政策扶持等措施，集中培育一批销售收入超30亿、50亿、100亿元的大型企业集团;筛选100个科技含量高、附加值高、市场前景好的重大工业结构调整项目，促其尽快建成投产。加快石油二机大型数字化钻机及配套开发、海洋钻机研制、南防年产3000台核电机、中源化工100万吨纯碱、时代矿业铜镍矿采选、淅铝技改扩能、汉冶特钢400万吨宽厚板升级改造、天池抽水蓄能电站等项目建设，力争完成年度投资200亿元以上。扎实做好南阳核电、白河南热电厂、宛西电厂、鸭电三期、邓州热电厂、裕丰复合肥厂等项目前期工作。大力发展中小企业。落实各项扶持政策，为中小企业创造宽松环境。发展多种层次中小企业信用担保体系，努力解决融资难问题。引导中小企业围绕龙头企业集聚发展。深入开展企业服务活动。实施"企业服务行动计划"，建立为企业服务的长效机制。实行经济运行例会制度，适时组织开展产销对接和银企洽谈活动。严格落实各项税费优惠政策，全面规范涉企收费，严惩"四乱"行为，切实减轻企业负担。加强煤电油运气供需调度，保证企业正常生产需要。整顿和规范市场秩序。重视加强企业家队伍建设，提高企业经营管理者素质。

壮大以现代服务业为主的第三产业。突出抓好文化、旅游、现代物流、金融等产业发展，促进现代制造业与服务业互动发展。深入挖掘开发汉文化、药文化、玉文化、商文化、盘古文化等特色文化，加快镇平玉文化改革发展试验区建设，实施一批文化产业重大项目，举办一批特色文化节会活动，创作一批文化精品。启动卧龙岗文化旅游产业集聚区规划建设，改造提升独山风景区，建设中心城区游客服务中心，打造南阳中心城区文化旅游品牌，强化中心城区旅游集散中心地位。强力实施十大旅游项目，打造精品景区，链接六大要素，提升服务质量，进一步打响伏牛山生态旅游和桐柏红色旅游等品牌，鼓励支持有实力的旅游公司组建大型旅游企业集团。积极开拓市场，加强旅游宣传促销。力争全年游客接待量达1600万人次以上，旅游综合收入达80亿元以上。修编实施我市现代物流业发展规划，加快建设区域性物流中心，集中培育一批大型现代物流企业和一批年交易额超百亿元的交易市场。继续实施万村千乡市场工程、双百市场工程和新网工程。发展新型商业业态，加快建设一批重点商圈、特色商业街、社区便民店。认真落实家电、汽车、摩托车、建材下乡和家电、汽车以旧换新等政策。加快金融业发展，支持民生银行、招商银行、浦发银行、兴业银行、广发银行等金融机构在我市设立分支机构，支持商业银行等地方金融机构发展壮大。以县域为单位组建农村商业银行、农村合作银行，积极发展村镇银行、小额贷款公司。推动政府投融资平台健康发展。优化金融生态环境，推进信用体系建设，力争全年各项贷款增长10%以上。推动3G网络和二代通信网络升级工程，促进信息化与工业化融合发展。进一步健全和落实稳定房地产市场的有关政策，按照支持居民合理住房消费、抑制投资投机性购房、增加有效供给、完善配套措施的原则，切实加大工作力度，促进房地产市场健康发展。重视扶持酒店业发展，繁荣餐饮市场。规范发展各类行业协会、商会等社会组织，充分发挥其在经济社会发展中的重要作用。

加快产业集聚区建设。坚持产城互动、融合发展，完善产业集聚区专项规划，出台实施促进产业集聚区发展的指导意见，增强产业集聚区承载力、吸引力和竞争力。加强集聚区水电路等基础设施建设，推广建设多层标准厂房。大力开展产业集聚区主题招商，加大项目引进建设力度，提高单位面积投资强度和产出效益。健全异地入区项目税收分享机制，引导老城区工业企业根据产业布局搬迁进入产业集聚区。健全产业集聚区投融资、中小企业担保、土地开发整理等机制，提升市场化运作能力。配套发展生产性服务业和生活性服务业，增强产业集聚区服务功能。探索建立产业集聚区管理运行机制、综合评价体系和考核制度，促其竞相发展、加速提升。

加强节能减排和生态环境建设。认真落实节能减排问责制和"一票否决制"。加快设备更新改造和重点节能工程建设。严格执行"两高一资"行业淘汰标准，加快淘汰落后产能，遏制水泥等行业重复建设。抓好市重点监管的96家耗能企业，积极开展建筑节能和交通节能，继续推进公共机构节能工作。推进环境容量预算，优化环境容量资源配置，完善环境自动监控体系。健全水环境预警制度，切实搞好饮用水水源保护，加强重点流域、重点区域、重点行业污染综合整治和重金属污染防治。认真抓好重点减排项目建设，加强对减排工程运行监管。加强林业生态建设，高标准启动实施环城高速绿化工程，全市完成造林60万亩。发展循环经济，加快工业固废资源化、农产品加工废弃物资源化等工程建设，全面推行清洁生产。加强土地资源管理，坚持最严格的耕地保护和节约用地制度。全面推进土地综合整治和城乡建设用地增减挂钩，千方百计保障重点项目用地。强化矿产资源开发监督管理，加快推进矿产资源整合，提高矿产资源开发利用率。

(二)统筹城乡协调发展，促进区域经济全面振兴

重视加强"三农"工作，加快城镇化进程，积极推进交通体系建设，促进城乡区域经济协调发展。

大力发展现代农业，致力改善农村生产生活条件。坚持把解决好"三农"问题作为重中之重，把增加农民收入、改善农村民生作为调整国民收入分配格局的重要内容，把扩大农村需求作为拉动内需的关键举措，把发展现代农业作为转变经济发展方式的重大任务。巩固提高粮食综合生产能力。扎实推进河南粮食生产核心区南阳主产区建设，实施"粮食稳定保收行动计划"，努力实现粮食总产稳定增长。落实好各项惠农补贴政策，保护和调动农民种粮积极性。提高科技投入和农机装备水平，挖掘农业增产潜力。加强农田水利基本建设。抓好19座病险水库除险加固、石步河水库新建、5万亩国家级现代灌区示范区建设和鸭灌、引丹等大中型灌区续建配套及节水改造，完成7.6万亩中低产田改造、5万亩标准农田建设和37万亩土地整理与复垦开发。调整优化农业结构。以"增量、提质、创牌、增效"为目标，推进优质农产品基地建设，重点建设大型商品粮、特色经济作物、畜产品、林产品、水产品生产基地，规划并启动10个高效农业集聚区建设。大力推进农业产业化，围绕每个农业特色产业，培育壮大一批龙头企业，提高农副产品就地加工转化能力，新发展农民专业合作社500家以上。积极发展休闲农业、乡村旅游和农村服务业。全面推进农村公共服务体系建设。完善农业科技服务体系、农村市场流通服务体系和农产品质量安全体系，新认证无公害农产品10个，全面启动县级农产品市场准入，在全市重点乡镇逐步开展畜产品市场准入工作。加强农村基础设施建设。启动农村邮政网络建设和新一轮农村电网改造，新解决25万农村居民饮水安全问题，新发展农村沼气用户3万户以上，建设县乡公路600公里、村道联网公路200公里、农村公路桥梁2000延米。推进农村环境治理，开工建设一批乡生活垃圾中转设施。认真做好扶贫开发新一轮十年规划工作，解决和巩固7万人以上脱贫问题。加快革命老区发展。深化"四议两公开"工作法，加强农村基层组织建设，完善村民自治机制，进一步规范村级民主选举、民主决策、民主管理、民主监督程序。做好新农村建设规划引导，合理布局，完善功能，完成600个中心村规划编制任务，新建100个新农村示范村、100个试点村。

做大做强中心城市。依托中原城市群，按照"白河为轴、两岸并举，一体两翼、区块突破，产业支撑、组团发展"的总体思路，围绕"聚集中心、加快中心、强化中心"，促进各种资源和要素向中心城市快速集聚，推进中心城市规模膨胀、实力壮大、品位提升。壮大城市综合经济实力。进一步做大做强中心城市装备制造、生物能源、生物化工、石油化工、电力、光电、食品等优势产业，培育壮大文化旅游、现代物流、金融保险、房地产业、总部经济等现代服务业，强化城市产业支撑。加快中心城区新能源和光电产业集聚区及东北分区发展步伐。加大高新区白河南区域基础设施建设和招商引资力度，积极争创国家级高新技术开发区。全面启动鸭河、官庄两个工区建设，界定发展范围，确定管理体制，编制各项规划，加强基础建设，制订部门帮扶措施，推动开发建设。修编南阳新区建设方案，争取尽快通过审批、启动实施。尽快完成白河景观带规划，加快推进两岸开发改造建设。高标准推进城市规划建设。完成城市总体规划审查报批工作，抓好重点区域控制性详规、修建性详规、各类专项规划编制和重点市政工程规划设计。增强规划透明度，加强对规划实施情况的监督检查，维护规划的严肃性、权威性。优化城区学校、医院布局，满足城市发展需要。搞好重点市政工程建设，新建、改造、续建城市道路22条，开工建设光武大桥，仲景大桥建成通车，改造背街小巷62条，完成所有旱厕改造，抓好污水处理厂二期工程和滨河路污水管网建设，加快白河南污水处理厂、新建垃圾处理场工程等项目运作，争取早日开工建设。积极推进旧城和城中村改造。搞好城市经营。坚持政府垄断土地一级市场，放开搞活二、三级市场，加大土地储备和闲置土地处置力度，不断增加土地储备规模。采取招标、拍卖、挂牌出让、股权转让、租赁、抵押、授权经营等方式盘活城市存量资产;大胆尝试造壳融资、借壳融资、发行企业债券、BOT等现代融资方式，搞活城市增量资本。坚持市县乡村四级联动，深入开展"六创一迎"。严格落实部门责任，全面完成"六城"年度创建任务。加大主要道路综合整治力度，深化市容环境卫生整治和交通秩序整治活动，开展规范化、专业化、制度化、精细化管理，构建完善的内部联动工作体系和快速反应机制，提升城市品位和外在形象。加大城市执法力度，集中清理整顿违法占地、违法建筑。加强市场建设与管理，坚决杜绝马路市场、店外经营、沿街促销等现象。进一步理顺城市管理体制，重视街道办事处和社区建设，开展市民素质教育，强化城市意识，充分调动各方参与支持城市管理的积极性。

积极推进县城和小城镇建设。大力实施"现代城镇体系建设工程"，突出产业为基、就业为本，增强城镇吸纳和辐射带动能力。培育壮大县级城市，以产业集聚区为载体，积极承接产业转移，促进特色产业、优势项目集聚发展，壮大县域经济，促进农民就近就业，尽快把县城发展成为常住人口20万人以上的中等城市，支持邓州加快建设成为南阳副中心城市。以"紧凑型"、"复合型"理念完成县域村镇体系规划，优化空间布局。继续开展争创"星级城镇"活动，突出抓好50强镇，大力发展特色小城镇和村级集镇。加快城镇道路、交通、供排水、燃气、绿化、污水及垃圾处理等基础设施建设和学校、医院、市场等公共设施建设，完善城镇功能。推进"六创一迎"向城镇延伸，提高城镇管理水平。加快户籍管理、社会保障制度改革，放宽城镇落户条件，支持在城镇稳定就业和居住的农民有序转变为城镇居民。

加快综合交通运输体系建设。启动"大交通"建设，以建设航空港、公路港、铁路港和信息港为重点，完善市域内高速公路网，兴建过境高速铁路客运专线和沿边县市出入省市的快速通道，加快构建各县市到达中心城区和南阳到达省会都市圈的对内对外双层1小时交通圈，逐步把我市建成中西部地区新的重要交通枢纽。加快南阳飞机场扩建和火车站站场改造工程，全力配合宁西铁路复线和郑渝高铁等重大工程建设。内邓高速公路完成年度投资9亿元，积极推进武西高速南阳段、侯十高速西坪至寺湾段前期工作。力争南阳至邓州、南阳至社旗一级公路早日开工。干线公路完成年度投资3.4亿元，农村公路完成年度投资3.2亿元。新建、续建公路运输场站13个。加强干线公路、县乡公路、村村通公路维修养护和车辆超限超载治理工作。坚持公交优先发展，加快公交场站建设，新投放城市公交车100辆，发展城际公交，再投放一定数量的出租车，方便群众出行。

(三)做好南水北调中线工程建设和第七届全国农运会筹备工作，提升经济社会发展水平

今年是南水北调中线工程建设和第七届全国农运会筹办的关键之年，必须迎难而上，抢抓机遇，全力推动，加快进度，以此带动全市经济社会发展整体提升。

抓好南水北调中线工程移民迁安和工程建设。南水北调中线工程对我市来说，重点在移民，难点也在移民。按照省委、省政府"四年任务两年完成"的总体要求和"搬得出、稳得住、能发展、可致富"的工作目标，扎实做好丹江口库区移民搬迁安置工作。第一批大规模移民搬迁安置工作，确保4月30日前完成移民新村建设，8月31日前完成生产安置和移民搬迁。大力推进第二批8.61万人移民迁安工作，实行交叉作业，3月底前做好移民安置对接工作，6月底前启动第二批移民新村建设，年底前房屋及基础设施基本完成，为明年4月前移民顺利搬迁奠定坚实基础。切实做好搬迁移民后期扶持工作。今年南水北调中线工程南阳段全线开工，积极搞好工程建设协调服务，全力以赴做好征地拆迁和群众安置工作，确保工程建设顺利推进。围绕水源地保护，加强库区生态建设，做好丹江口库区水污染防治和水土保持规划修编及经济社会发展规划编制工作，积极争取国家生态建设补偿资金和受水区对口支援，促进我市经济社会健康发展。

扎实做好农运会各项筹备工作。进一步加大宣传力度，统一思想，提高认识，全民动员，着力营造人人参与支持农运会筹办的浓厚氛围。全面加快场馆建设，完成比赛项目和接待设施布局工作，启动迎宾馆、代表团驻地、运动员村建设，确保综合训练馆、游泳馆年底前竣工、拆迁安置房按期交付群众入住。加快主体育场、新闻中心、全民健身广场、县市区和高校比赛场馆及接待设施建设维修进度，确保按时完工。组织好第七届农运会北京新闻发布会暨吉祥物、主题口号全国征集活动。增强机遇意识，积极争取国家和省政策资金支持。全面启动筹资工作，大力推进市场开发和社会捐赠工作，确保农运会资源开发工作取得较大进展。

(四)积极推进改革开放，增强加快发展的动力活力

深入推进各项改革。实施"重点改革行动计划"。完成市属国有工业企业改革遗留工作，帮助改制企业尽快恢复生产。全面推进国有流通企业改革。积极实施"421"企业上市方案，力争今年有2-3家企业上市融资。加快行政管理体制改革，如期完成市县政府机构改革。继续深化财税体制改革、事业单位分类改革和人事制度改革。认真落实义务教育、公共卫生、基层医疗卫生事业单位绩效工资政策。继续做好公务员津贴补贴规范和兑现工作，市级年内落实到位，县级两年内落实到位。推进行政事业单位资产运营改革，加强对政府经营性投资项目和公司的监督管理，防止国有资产收入流失，确保国有资产保值增值。深化投资体制改革，政府投资的非经营性项目全面推行代建制。加快医药卫生体制改革，促进基本公共卫生服务均等化。深化农村综合改革，稳定土地承包经营政策，开展土地流转市场改革试点;完成集体林权制度主体改革任务，启动配套改革;基本完成农村义务教育债务化解工作;推进农村公益事业建设"一事一议"财政奖补试点。加快推动文化体制、县级供销社、资源价格、金融、环保等领域的改革。

进一步扩大对外开放。把招商引资作为带动全局的战略性任务，实施"对外开放行动计划"，深入开展大招商活动，努力夺取招商引资工作新胜利。进一步落实招商责任，突出招商重点，创新招商方式，注重招商质量和实效，加强签约项目的跟踪落实，强化落地项目服务，力争签约合同引资额超过600亿元，实际利用外资1.8亿美元，实际到位省外资金260亿元。高水平组织第八届玉雕节和第九届张仲景医药科技文化节，积极参加或举办各类境内外大型经贸洽谈活动。加强与中国兵器集团、兵装集团、中核总、中建材、首钢控股、内蒙古博源、乐凯、平安、恒天集团等大企业的战略合作，加快落实天冠与国开投、中光学与四川长虹、南防与平安集团战略合作协议。加大扶持力度，培育一批主业突出、核心竞争力强的出口龙头企业。争取设立南阳出口加工保税区，方便外商投资，提供出口便利，提高南阳对外开放知名度。加快5个省对外开放重点县市、3个省商务工作扩权县市对外开放步伐，带动县域开放型经济发展。

(五)大力实施民生工程，促进社会和谐稳定

加大民生保障和改善力度，实施好"十项民生工程"，让人民群众更多地享受到改革发展的成果，保持社会和谐稳定。

做好就业和社会保障工作。坚持积极的就业政策，大力实施"就业促进行动计划"。继续落实好"五缓四减三补"政策，帮助困难企业和破产改制企业稳定扩大就业、减少失业。加强对高校毕业生就业培训指导。做好复转军人就业安置工作。支持自主创业、自谋职业，改善创业环境，以创业带动就业，发放小额担保贷款1亿元以上。全年新增城镇就业8万人以上，下岗失业人员再就业2.5万人以上，其中"4050"等就业困难人员1万人，"零就业家庭"动态归零，新增农村劳动力转移就业10万人以上。重视解决失地农民就业和社会保障问题。提高企业退休人员基本养老金和部分优抚对象待遇。强力推进社会保险扩面征缴清欠工作，新增城镇企业职工基本养老保险1.2万人，城镇居民基本医疗保险稳定在65万人以上。加快医疗保险市级统筹步伐，提高城镇职工和居民基本医保最高支付限额标准和住院支付比例，基本解决关闭破产企业退休人员和困难职工基本医疗保障问题，将新农合财政补助标准提高到120元。抓好国家首批新型农村养老保险试点工作，3个试点县力争参保人数达80万人以上。进一步完善城乡低保制度，提高农村五保供养水平。大力发展残疾人事业、社会福利和慈善事业。全面推进住房公积金制度，新建续建经济适用房56.8万平方米，新增廉租房8725套，积极推进棚户区改造。

加快发展社会事业。加大教育投入，加强薄弱学校和师资队伍建设，建立教师队伍长效补进机制。强力推进义务教育均衡发展，努力推进教育公平，尽最大可能满足人民群众对优质教育资源的需求。完成农村中小学校舍维修改造10万平方米，改扩建、资源整合中心城区中小学校8所。高度重视高中特别是农村高中教育，让更多的适龄人口能够接受高中阶段教育。大力实施职教攻坚计划，推进民办职业教育发展，提高劳动者素质，为南阳经济发展培养实用人才。积极推进南阳医专升本和河南经济管理学校、南阳农校、幼师、艺校升专工作，启动镇平玉雕职业技术学院建设。抓好高层次创新型人才队伍建设，实施"人力资源素质提升行动计划"，开辟"绿色通道"吸引优秀人才到我市工作。加大文化事业投入，抓好50个乡镇文化站、200个村级文化大院建设，完成481个村广播电视村村通和5万场次农村电影放映任务。加强城乡卫生服务体系建设，完成27个乡镇卫生院、600个标准化卫生所和5个社区服务中心建设任务，从市县两级选派100名医师对口支援50家乡镇卫生院。扶持和促进中医药事业发展。继续实施国家免疫规划，对农村妇女进行免费宫颈癌检查，努力实现全市农村妇女住院分娩(平产)免费。筹建规模更大、功能更全的市老干部活动中心。精心做好省十一届运动会备战工作。把贯彻落实《全民健身条例》与筹办第七届全国农运会紧密结合起来，打造全国全民健身试点城市。深化人口和计划生育综合改革，稳定低生育水平，提高出生人口素质。加强统计工作，认真做好第六次全国人口普查。

全力维护社会和谐稳定。实施"安全发展行动计划"，严格落实安全生产责任制，深入开展安全生产隐患排查治理工作，强化监督管理，坚决遏制重特大安全事故发生。深入推进社会管理创新，加强和改进信访工作，拓宽群众利益诉求表达渠道，提升社会矛盾纠纷化解能力，形成依法有序表达诉求、及时有效解决问题的社会环境。坚决纠正损害群众利益的突出问题，妥善处理土地征用、拆迁安置、企业改制、环境污染、劳资纠纷、涉法涉诉等方面的矛盾和问题。加强地震监测台网等防灾减灾基础建设，加强应急管理，积极预防和妥善处置群体性事件及突发公共安全事件。深入开展平安创建活动，依法严厉打击各类违法犯罪活动，完善社会治安防控体系，强化社会治安综合治理，提高群众安全感和满意度。加强食品药品质量监管。积极开展法律服务和法律援助。深入推进社会主义核心价值体系建设，加强诚信教育和文明礼仪教育，广泛开展精神文明创建活动。

同时，继续加强国防教育和国防后备力量建设，支持驻宛解放军和武警部队建设，不断提高预备役部队、民兵和人民防空建设质量;深入开展双拥共建活动，巩固发展军政军民团结，积极创建国家级双拥模范城。切实做好物价、外事、侨务、对台、民族、宗教、档案、保密、史志、气象等工作。超前谋划，编制好"十二五"发展规划
